# Male sexual dysfunction in obesity: The role of sex hormones and small fibre neuropathy

**DOI:** 10.1371/journal.pone.0221992

**Published:** 2019-09-11

**Authors:** Jan Hoong Ho, Safwaan Adam, Shazli Azmi, Maryam Ferdousi, Yifen Liu, Alise Kalteniece, Shaishav S. Dhage, Brian G. Keevil, Akheel A. Syed, Basil J. Ammori, Tomás Ahern, Rachelle Donn, Rayaz A. Malik, Handrean Soran

**Affiliations:** 1 Department of Medicine, Manchester University NHS Foundation Trust, Manchester, United Kingdom; 2 Cardiovascular Research Group, The University of Manchester, Manchester, United Kingdom; 3 Department of Biochemistry, Wythenshawe Hospital, Manchester University NHS Foundation Trust, Manchester, United Kingdom; 4 Department of Diabetes & Endocrinology, Salford Royal NHS Foundation Trust, Salford, United Kingdom; 5 Department of Surgery, Salford Royal NHS Foundation Trust, Salford, United Kingdom; 6 Department of Endocrinology, Our Lady of Lourdes Hospital, RCSI Hospital Group, Drogheda, Ireland; 7 Department of Medicine, Weill Cornell Medicine-Qatar, Doha, Qatar; University of Porto, PORTUGAL

## Abstract

**Context:**

Multiple factors contribute to sexual dysfunction in men with obesity. Sex hormone levels are commonly abnormal in men with obesity and this abnormality is often the focus of management in clinical practice. The role of small fibre neuropathy in obesity-related sexual dysfunction is not well established.

**Objective:**

We aimed to investigate the relationship between sexual function, sex hormone levels and small nerve fibre morphology in men with severe obesity.

**Materials and methods:**

A prospective study of 29 men with severe obesity was undertaken. Sexual function was assessed using the European Male Ageing Study Sexual Function Questionnaire. Small nerve fibre morphology was quantified using corneal confocal microscopy. Sex hormone levels were measured by mass spectrophotometry.

**Results:**

Erectile dysfunction was present in 72% of the cohort with a higher prevalence of diabetes among the symptomatic group (88% vs 38%, p = 0.006). Corneal nerve fibre length (CNFL) and corneal nerve fibre density (CNFD) were both significantly lower in participants with erectile dysfunction compared to those without (p = 0.039 and p = 0.048 respectively). The erectile function score correlated with CNFL (r = -0.418, p = 0.034) and CNFD (r = -0.411, p = 0.037). Total testosterone and calculated free testosterone levels did not differ significantly between men with or without erectile dysfunction (median 8.8 nmol/L vs 9.0 nmol/L, p = 0.914; and median 176 pmol/L vs 179 pmol/L, p = 0.351 respectively), infrequent sexual thoughts (median 8.1 nmol/L vs 9.2 nmol/L, p = 0.650; and median 184 pmol/L, vs 176 pmol/L, p = 0.619 respectively) and decreased morning erections (median 9.0 nmol/L vs 8.8 nmol/L, p = 0.655; and median 170 pmol/L vs 193 pmol/L, p = 0.278 respectively).

**Conclusion:**

Sexual dysfunction is highly prevalent in men with severe obesity. We found an association between small fibre neuropathy with erectile dysfunction with presence of diabetes a likely a significant contributing factor. We found no associations between testosterone levels with sexual symptoms (including frequency of sexual thoughts). The influence of small nerve fibre neuropathy on response to therapeutic interventions and whether interventions that improve small fibre neuropathy can improve erectile function in this population merits further study.

## Introduction

Sexual dysfunction is common in men with obesity [1]. Normal sexual functioning is dependent on complex vascular, neural, hormonal and psychological factors, all of which are potentially affected by obesity [2, 3]. Low testosterone level, in particular, is thought to have a bidirectional relationship with obesity [4, 5] and is common in men with obesity [6] and is very common in men with severe obesity [7, 8]. Current guidelines recommend testosterone replacement in men with symptomatic androgen deficiency to improve general well-being, bone mineral density and sexual function [9]. Whilst testosterone therapy may improve sexual symptoms in hypogonadal men [9], evidence that it benefits sexual function in men with obesity and low levels of testosterone is inconsistent [10–13].

Erectile dysfunction is associated with diminished small and large nerve fibre sensory thresholds [14]. Peripheral neuropathy, especially small fibre neuropathy, occurs more commonly in people with obesity than in those without [15, 16]. Corneal confocal microscopy is a rapid, validated and non-invasive technique that assesses small nerve fibre integrity [17, 18] with comparable diagnostic efficiency to intraepidermal nerve fibre density in diabetic peripheral neuropathy [19]. We have recently shown that small fiber neuropathy, quantified using corneal confocal microscopy, is associated with erectile dysfunction in men with type 1 diabetes [20].

We aimed to assess, for the first time, whether relationships exist between sexual symptoms with small fibre neuropathy and/or with sex hormone levels in men with severe obesity.

## Materials and methods

### Participants

Twenty-nine male patients with severe obesity were recruited from the weight management clinic at Salford Royal National Health Service Foundation Trust (Salford, United Kingdom). Severe obesity is defined as BMI above 40 which is equivalent to obesity class III using the BMI classification set out by the World Health Organisation (WHO) [21]. Patients known to have cardiovascular disease, disease of the pituitary, testes or adrenal glands, or taking therapy for erectile dysfunction or known to affect androgen levels or cause erectile dysfunction and luteinising hormone >9.4 U/L (primary hypogonadism), were excluded [22]. Further exclusion criteria included: other causes of peripheral neuropathy apart from diabetes; cancer, radiotherapy or chemotherapy; previous eye surgery and corneal disease. Presence of comorbidities such as hypertension and type 2 diabetes were determined on medical history. A HbA1c measurement at baseline was used in addition to identify patients with undiagnosed type 2 diabetes (HbA1c ≥ 48 mmol/mol) and pre-diabetes (HbA1c 42–47 mmol/mol) [23]. This study has approval from the Greater Manchester Central Research and Ethics Committee. Written informed consent was obtained from all individuals prior to participation.

### Sexual function assessment

Sexual function was assessed using the European Male Ageing Study Sexual Function Questionnaire [24]. Three sexual symptoms were used for assessment of sexual function: erectile function, frequency of sexual thoughts, and frequency of morning erections. Participants were divided into symptomatic and asymptomatic groups using previously established cut-offs based on validated scores for each individual question relating to the three sexual symptoms [25, 26].

### Neuropathy assessment

Symptoms of peripheral neuropathy was assessed using the neuropathy symptom profile (NSP). Cold (CT) and warm (WT) perception thresholds were assessed on the dorsolateral aspect of the left foot (S1) using the TSA-II NeuroSensory Analyser (Medoc, Ramat-Yishai, Israel). Electrodiagnostic studies were undertaken using a Dantec Keypoint system (Dantec Dynamics, Bristol, UK). Vibration perception threshold (VPT) was established using a Horwell Neurothesiometer (Scientific Laboratory Supplies, Wilfrod, Nottingham, UK). Deep breathing heart rate variability (DB-HRV) was established using an ANX 3.0 autonomic nervous system monitoring device (ANSAR Medical Technologies, Philadelphia, PA, USA).

### Corneal confocal microscopy

Corneal nerve morphology was assessed using corneal confocal microscopy [17, 27]. Corneal confocal microscopy (Heidelberg Retinal Tomograph III Rostock Cornea Module; Heidelberg Engineering, Heidelberg, Germany) was performed using our established protocol [27]. Six non-overlapping images from the centre of the cornea were selected per patient (three per eye) [28]. Automated image analysis was performed using ACCMetrics software (The University of Manchester, United Kingdom). Corneal nerve fibre length (total length of nerves in mm per mm^2^), corneal nerve fibre density (CNFD) (number of major nerves per mm^2^) and corneal nerve branch density (CNBD) (number of nerve branches per mm^2^) were quantified [29].

### Laboratory measurements

Venous blood samples were obtained between the hours of 0800 and 1100 after an overnight fast of at least 12 hours. Apart from glycosylated haemoglobin (HbA1c) which was measured using standard laboratory methods in the Department of Biochemistry, Manchester University NHS Foundation Trust on the day of collection, all other laboratory measurements were performed at the end of the study. Serum or plasma, isolated within 2 hours of collection, was stored at 4°C or -20°C until analysed. Each serum aliquot was stored for a maximum of two years and underwent one freeze-thaw cycle only. Serum total testosterone, dihydrotestosterone, dehydroepiandrosterone sulphate and androstenendione levels were determined using liquid chromatography–tandem mass spectrometry in a validated clinical laboratory [30, 31]. Sex hormone-binding globulin, luteinising hormone and follicle-stimulating hormone levels were measured electrochemiluminescence immunoassay (Roche Diagnostics) using Roche^®^ automated analysers (E170 platform). Serum free testosterone levels were calculated using the mass action equation described by Vermeulen [32] and participant-specific total testosterone, sex hormone-binding globulin and albumin levels. A participant was considered to have a low testosterone level if either; his total testosterone level was less than 8 nmol/L; or his total testosterone level was between 8 and 11 nmol/L *and* his calculated free testosterone level was less than 220 pmol/L [25].

### Statistical analyses

All statistical analyses were performed using SPSS for Windows (Version 23.0, IBM SPSS Statistics, Armonk, NY). Continuous variables were compared between groups using the independent samples t-test or, in the case of non-parametrically distributed data, the Mann-Whitney U test. Normality of data distribution was assessed for all continuous variables using the Shapiro-Wilk test. The chi-squared test was used for analysis of categorical data. Correlations between variables were assessed using Spearman’s analyses. Results are expressed as mean with standard deviation (SD) for parametric data and as median with interquartile range (IQR) for non-parametric data. No attempt was made to adjust for missing data. The level of statistical significance was set at less than 0.05 for all analyses.

## Results

Sixteen (55%) of the participants had erectile dysfunction of whom 34% had severe erectile dysfunction. Infrequent sexual thoughts and decreased morning erections were reported in 13 (45%) and 23 (79%) of the participants respectively. The median overall satisfaction score corresponded with moderate dissatisfaction.

The median total testosterone was 9.0 nmol/L, 95% confidence interval ranged between 7.4 nmol/L and 11.1 nmol/L. The total testosterone level was less than 8 nmol/L in 12 (41%) participants, between 8 and 11 nmol/L in ten (34%) participants, of whom 4 (14%) had a calculated free testosterone level below 220 pmol/L.

### Erectile dysfunction

When participants were divided into symptomatic and asymptomatic groups based on their erectile function scores, age was higher and body mass index was lower in the symptomatic group (Table 1). The prevalence of type 2 diabetes mellitus and hypertension were higher in those with erectile dysfunction. The HbA1c, however, did not differ significantly between the groups. The prevalence of dysglycaemia (pre-diabetes and type 2 diabetes mellitus) also did not differ between symptomatic and asymptomatic groups.

**Table 1:** Comparison of demographics, clinical characteristics, and sex hormone levels between asymptomatic and symptomatic patients divided based on erectile function.

	Asymptomatic based on erectile function (n = 13)	Symptomatic based on erectile function (n = 16)	p-value
**Clinical characteristics**			
**Age, years**	44.4 ±8.4	52.1 ±10.9	**0.045**
**BMI, kg/mm**^**2**^	54.8 ±12.4	46.9 ±7.3	**0.041**
**Type 2 diabetes, n (%)**	5 (38%)	14 (88%)	**0.006**
**Duration of diabetes, years**	5±3	6±5	0.787
**Pre-diabetes and type 2 diabetes, n (%)**	10 (77%)	14 (88%)	0.453
**Hypertension, n (%)**	4 (30%)	11 (68%)	**0.042**
**Antihypertensives, n**	0 (0–1)	1 (0–2)	0.092
**Biochemistry**			
**HbA1c, mmol/mol**	52±13	55±15	0.591
**Total cholesterol, mmol/l**	3.8±1.2	4.0±1.0	0.796
**Triglyceride, mmol/l**	1.0±0.5	1.3±0.5	0.206
**HDL-C, mmol/l**	1.01±0.38	0.95±0.22	0.702
**LDL-C, mmol/l**	2.5±1.0	2.4±0.8	0.808
**Sex hormones**			
**Low testosterone, n (%)**	7 (54)	10 (63)	0.638
**Total testosterone, nmol/L**	9.0 (6.4–12.3)	8.8 (6.4–11.0)	0.914
**Free testosterone, pmol/L**	179 (132–311)	176 (120–216)	0.351
**Sex hormone-binding globulin, nmol/L**	29.2 (21.7–35.5)	32.1 (21.7–38.3)	0.559
**Luteinising hormone, mIU/Ml**	2.3±1.2	3.5±1.9	0.059
**Follicle-stimulating hormone, mIU/L**	4.2 (2.9–4.7)	3.5 (2.4–4.9)	0.779
**Dihydrotestosterone, nmol/L**	0.60 (0.34–0.98)	0.62 (0.50–0.99)	0.914
**Dehydroepiandrosterone sulphate, nmol/L**	2.2 (1.1–3.4)	1.3 (0.9–3.6)	0.537
**Androstenedione, nmol/L**	2.3 (1.6–3.0)	1.6 (1.3–2.4)	0.170

**Notes:** Data are presented as mean and standard deviation for normally-distributed variables and median and interquartile range for non-parametric variables. Independent t-test was performed for normally-distributed variables, Mann-Whitney U test for non-parametric variables, and chi-squared test for categorical variables when comparing asymptomatic and symptomatic groups. p<0.05 is considered statistically significant.

**Abbreviations:** BMI, body mass index; HbA1c, glycosylated haemoglobin; HDL-C, high-density lipoprotein cholesterol; LDL-C, low-density lipoprotein cholesterol.

**Fig 1:**
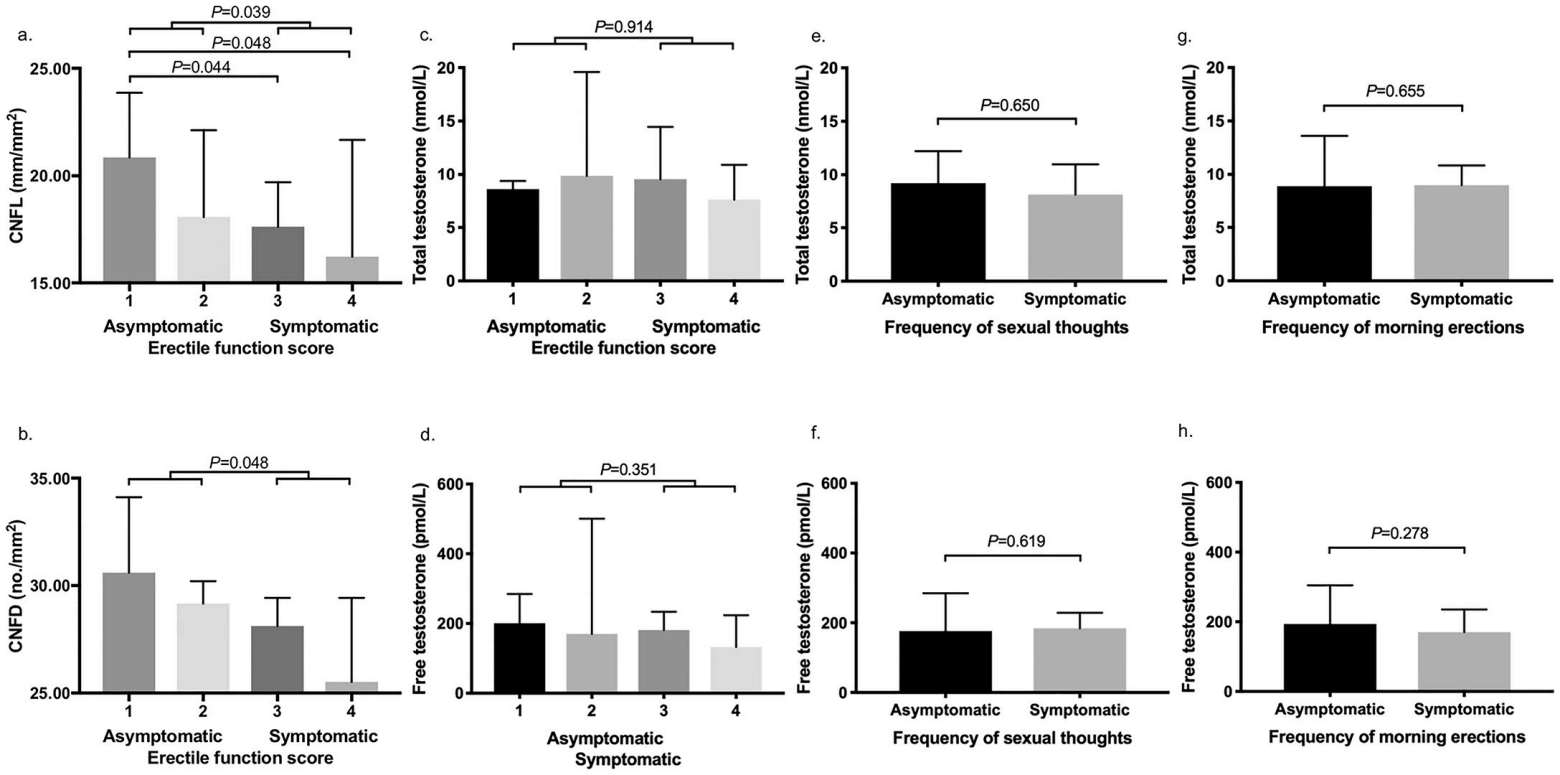
Comparison of corneal nerve parameters in asymptomatic and symptomatic patients based on erectile function score (a & b) and comparison of total and free testosterone levels between asymptomatic and symptomatic patients based on erectile function (c & d), frequency of sexual thoughts (e & f), and frequency of morning erections (g & h). CNFL and CNFD are both significantly lower in symptomatic compared to asymptomatic patients with erectile dysfunction (a & b). Data are presented as mean and standard deviation for normally-distributed variables and median and interquartile range for non-parametric variables. Erectile function questionnaire response categories: 1: always able to keep erection good enough for sexual intercourse, 2: usually able, 3: sometimes able, 4: never able. Abbreviations: CNFD, corneal nerve fibre density; CNFL, corneal nerve fibre length. p<0.05 is considered statistically significant.

Both CNFL and CNFD were lower in participants with erectile dysfunction compared to those without (Table 2 and Fig 1). Erectile function score correlated with both CNFL (Spearman’s r = -0.418, p = 0.034) and CNFD (Spearman’s r = -0.411, p = 0.037).

**Table 2:** Comparison of measures of neuropathy between asymptomatic and symptomatic patients divided based on erectile function.

	Asymptomatic based on erectile function (n = 13)	Symptomatic based on erectile function (n = 16)	p-value
**NSP, /38**	0 (0–5)	4 (0–15)	0.131
**VPT, V**	13.3±5.6	16.3±7.2	0.296
**CT, °C**	22.6±5.9	20.6±8.2	0.528
**WT, °C**	41.0±2.3	41.9±3.3	0.437
**CNFL, mm/mm**^**2**^	20.29±3.21	16.74±4.45	**0.039**
**CNFD, no./mm**^**2**^	30.21 (27.34–33.59)	27.60 (22.50–29.17)	**0.048**
**CNBD, no./mm**^**2**^	60.75±33.53	45.70±24.86	0.201
**DB-HRV, beats/min**	35 (24–44)	14 (12–23)	**0.016**

**Notes:** Data are presented as mean and standard deviation for normally-distributed variables and median and interquartile range for non-parametric variables. Independent t-test was performed for normally-distributed variables, Mann-Whitney U test for non-parametric variables, and chi-squared test for categorical variables when comparing asymptomatic and symptomatic groups. p<0.05 is considered statistically significant.

**Abbreviations:** CNFD, corneal nerve fibre density; CNBD, corneal nerve branch density; CNFL, corneal nerve fibre length; CT, cold perception threshold; DB-HRV, deep breathing heart rate variability; NSP, neuropathy symptom profile; VPT, vibration perception threshold; WT, warm perception threshold.

The level of total testosterone, free testosterone, sex hormone-binding globulin, dihydrotestosterone, dehydroepiandrosterone sulphate and androstenendione did not differ between participants with and without erectile dysfunction (Table 1 and Fig 1). There was no difference in the prevalence of low testosterone between these two groups. Total and free testosterone did not correlate with erectile function (Spearman’s r = -0.083, p = 0.667 and Spearman’s r = -0.255, p = 0.181 respectively).

There were no difference in lipid profile between the two groups.

### Infrequent sexual thoughts and decreased morning erections

Clinical, corneal nerve or hormone parameters did not differ between those with and without infrequent sexual thoughts (S2 Table) or in those with and without decreased morning erections (S3 Table). Total and free testosterone did not correlate with frequency of sexual thoughts (Spearman’s r = 0.236, p = 0.218 and Spearman’s r = 0.179, p = 0.352 respectively) or with frequency of morning erections (Spearman’s r = 0.079, p = 0.682 and Spearman’s r = 0.186, p = 0.333 respectively). The HbA1c and lipid profile did not differ between patients with and without infrequent sexual thoughts and patients with and without decreased morning erections (S2 and S3 Tables).

### Low testosterone

There was no difference in sexual symptom frequency between men with and without a low testosterone level (S4 Table).

## Discussion

This is the first study to assess sexual function, small fibre neuropathy, sex hormone levels and their relationships simultaneously in men with severe obesity. Male sexual dysfunction was highly prevalent in our study population with symptomatic erectile dysfunction and reduction in frequency of sexual thoughts being present in 55% and 45% of patients, respectively. The vast majority of patients had overall sexual function scores below the mean value found in the European Male Ageing Study, despite being ten years younger (mean age of 49 compared to 59 years) [24]. The level of distress related to sexual functioning was also higher compared to that observed in the European Male Ageing Study cohort.

We have demonstrated, for the first-time, that reduction in small nerve fibre indices occur in patients with severe obesity and erectile dysfunction. In particular, corneal nerve fibre density and length were significantly lower in patients with symptomatic erectile dysfunction and they correlated with erectile function. There was, however, no difference between corneal nerve branch density and the severity of erectile dysfunction. We hypothesise that this could be a result of constant changes in this parameter secondary to nerve regeneration. There was also evidence of autonomic dysfunction in patients with erectile dysfunction. Older age, obesity and a higher prevalence of diabetes and hypertension in the cohort with erectile dysfunction are likely to be the major contributors of corneal nerve loss in accord with our previous studies [33, 34] and our recent study in type 1 diabetes.

Although somatic and autonomic neuropathy are associated with erectile dysfunction [35], patients continue to have assessment of sex hormones and cardiovascular risk factors, but not neuropathy. Our study emphasizes the importance of assessing small fibre neuropathy as it plays an important role in the neurovascular regulation of erectile function [36, 37]. Although phosphodiesterase type 5 inhibitors (PDE5i) are used in the management of erectile dysfunction, they are less effective in people with nerve damage [38, 39]. Based on our study findings, we therefore suggest that assessment of small fibre neuropathy prior to initiation of PDE5i may help identify patients who are more likely to respond to treatment and corneal confocal microscopy provides a rapid, objective and clinically feasible method to assess small fibre damage and repair [17–19]. Larger studies will be required to prove this further.

In keeping with previously published studies, we found a high prevalence of low testosterone levels in this cohort of men with severe obesity [8]. However, testosterone levels were not associated with sexual symptoms and sexual symptom frequency did not differ between those with low and normal testosterone levels. This perhaps suggests that low testosterone might not be a major determinant of presence of sexual symptoms in severe obesity.

The limitations of this study are the cross-sectional design which limits the inference of cause and effect between small fibre neuropathy and erectile dysfunction. A larger sample size would have allowed adjustment of confounding factors for small fibre damage in relation to erectile dysfunction. A single sample to determine sex hormone levels may also be seen as a limitation, however, several studies have confirmed that testosterone levels do not fluctuate significantly when measured serially in a timed sample over several months [40–42].

## Conclusion

We conclude that erectile dysfunction is common among men with severe obesity and is associated with small fibre neuropathy, likely to be further driven by older age, diabetes and cardiovascular risk factors. Corneal confocal microscopy is a rapid, non-invasive technique to quantify of small nerve fibre degeneration and regeneration. Prospective studies are required to assess the impact of small fibre neuropathy on the effectiveness of therapies for erectile dysfunction and whether interventions that improve small fibre neuropathy can improve erectile dysfunction in men with severe obesity.

## Supporting information

S1 Table Questions regarding sexual symptoms within the EMAS sexual function questionnaire and definitions of asymptomatic and symptomatic response categories. The definitions of asymptomatic and symptomatic response categories are based on validated published criteria [25, 26].
(DOCX)

S2 Table Comparison of sex hormone levels between asymptomatic and symptomatic patients divided based on frequency of sexual thoughts. **Notes:** Data are presented as mean and standard deviation for normally-distributed variables and median and interquartile range for non-parametric variables. Independent t-test was performed for normally-distributed variables, Mann-Whitney U test for non-parametric variables, and chi-squared test for categorical variables. p<0.05 is considered statistically significant. **Abbreviations:** CNFD, corneal nerve fibre density; CNBD, corneal nerve branch density; CNFL, corneal nerve fibre length.
(DOCX)

S3 Table Comparison of sex hormone levels between symptomatic and asymptomatic patients divided based on frequency of morning erections. **Notes:** Data are presented as mean and standard deviation for normally-distributed variables and median and interquartile range for non-parametric variables. Independent t-test was performed for normally-distributed variables, Mann-Whitney U test for non-parametric variables, and chi-squared test for categorical variables. p<0.05 is considered statistically significant. **Abbreviations:** CNFD, corneal nerve fibre density; CNBD, corneal nerve branch density; CNFL, corneal nerve fibre length.
(DOCX)

S4 Table Comparison of sexual symptoms between groups with low testosterone and normal testosterone. Data are presented as median and interquartile range for non-parametric variables. Mann-Whitney U test was performed for non-parametric variables. Questionnaire response categories: erectile function: 1: always able to keep erection good enough for sexual intercourse, 2: usually able, 3: sometimes able, 4: never able; frequency of sexual thoughts and morning erection frequency: 1: none or once in the past month, 2: 2–3 times/month and 1 time/week, 3: 2–6 times/week, 4: ≥1/day; overall satisfaction: 0: very dissatisfied, 1: moderately dissatisfied, 2: equally satisfied and dissatisfied, 3: moderately satisfied, 4: very satisfied. Overall sexual function score ranges from 0 to 33 with higher scores corresponding with higher level of sexual functioning. Sexual functioning-related distress ranges from 0 to 20, with higher scores corresponding with higher level of distress. p<0.05 is considered statistically significant.
(DOCX)
