# Supplementary material for: Male sexual dysfunction in obesity: The role of sex hormones and small fibre neuropathy
Source: PLoS One. 2019 Sep 11;14(9):e0221992. doi: 10.1371/journal.pone.0221992 (PMC6738611; doi:10.1371/journal.pone.0221992)
Supplement: S1 Table — The definitions of asymptomatic and symptomatic response categories are based on validated published criteria [25, 26]. (DOCX) [file pone.0221992.s001.docx]

**S1 Table.** Questions regarding sexual symptoms within the EMAS sexual function questionnaire and definitions of asymptomatic and symptomatic response categories.

|  | Asymptomatic | Symptomatic |
| --- | --- | --- |
| Were you able to get and keep an erection sufficient for sexual intercourse? | Usually or always | Never or sometimes |
| How often did you think about sex? | Once a week or more | 2–3 times in the past month |
| How frequently did you awaken with a full erection in the past month? | 2–3 times in the past month | ≤1 time in the past month |

The definitions of asymptomatic and symptomatic response categories are based on validated published criteria.
