# Supplementary material for: Male sexual dysfunction in obesity: The role of sex hormones and small fibre neuropathy
Source: PLoS One. 2019 Sep 11;14(9):e0221992. doi: 10.1371/journal.pone.0221992 (PMC6738611; doi:10.1371/journal.pone.0221992)
Supplement: S2 Table — Notes: Data are presented as mean and standard deviation for normally-distributed variables and median and interquartile range for non-parametric variables. Independent t-test was performed for normally-distributed variables, Mann-Whitney U test for non-parametric variables, and chi-squared test for categorical variables. p<0.05 is considered statistically significant. Abbreviations: CNFD, corneal nerve fibre density; CNBD, corneal nerve branch density; CNFL, corneal nerve fibre length. (DOCX) [file pone.0221992.s002.docx]

**S2 Table**. Comparison of sex hormone levels between asymptomatic and symptomatic patients divided based on **frequency of sexual thoughts**.

|  | Asymptomatic based on frequency of sexual thoughts  (n=16) | Symptomatic based on frequency of sexual thoughts  (n=13) | *P*-value |
| --- | --- | --- | --- |
| Clinical characteristics |  |  |  |
| Age, years | 45.9±10.2 | 52.0±10.0 | 0.122 |
| Body mass index, kg/mm^2^ | 51.4±11.1 | 49.3±9.7 | 0.613 |
| Type 2 diabetes, n (%) | 9 (56%) | 14 (76%) | 0.244 |
| Duration of diabetes, years | 5±4 | 6±4 | 0.716 |
| Hypertension, n (%) | 8 (50%) | 7 (54%) | 0.837 |
| Antihypertensives, n | 0 (0–1) | 1 (0–2) | 0.983 |
| Biochemistry |  |  |  |
| HbA1c, mmol/mol | 49 (42–61) | 51 (42–60) | 0.982 |
| Total cholesterol, mmol/l | 3.5±0.8 | 4.1±1.0 | 0.173 |
| Triglyceride, mmol/l | 1.1±0.6 | 1.3±0.4 | 0.256 |
| HDL-C, mmol/l | 1.03±0.38 | 0.94±0.20 | 0.499 |
| LDL-C, mmol/l | 2.0±0.5 | 2.5±0.9 | 0.195 |
| Sex hormones |  |  |  |
| Low testosterone, n (%) | 10 (63%) | 7 (54%) | 0.716 |
| Total testosterone, nmol/L | 9.2 (6.9–12.2) | 8.1 (5.5–11.0) | 0.650 |
| Free testosterone, pmol/L | 176 (135–285) | 184 (107–229) | 0.619 |
| Sex hormone-binding globulin, nmol/L | 32.1 (24.1–38.7) | 28.0 (20.6–33.5) | 0.374 |
| Luteinising hormone, mIU/mL | 3.0±1.9 | 3.1±1.7 | 0.930 |
| Follicle-stimulating hormone, mIU/L | 3.5±1.8 | 3.7±1.3 | 0.720 |
| Dihydrotestosterone, nmol/L | 0.61 (0.39–1.03) | 0.60 (0.50–0.85) | 0.650 |
| Dehydroepiandrosterone sulphate, nmol/L | 2.2 (1.1–3.4) | 1.2 (0.8–3.9) | 0.537 |
| Androstenedione, nmol/L | 2.4 (1.6–3.1) | 1.6 (1.3–2.2) | 0.050 |
| Corneal nerve parameters |  |  |  |
| CNFL, mm/mm^2^ | 18.41±3.93 | 17.81±4.82 | 0.733 |
| CNFD, no./mm^2^ | 28.12 (26.04–30.21) | 28.12 (22.92–31.77) | 0.880 |
| CNBD, no./mm^2^ | 49.85±33.00 | 53.12±25.23 | 0.779 |

**Notes:** Data are presented as mean and standard deviation for normally-distributed variables and median and interquartile range for non-parametric variables. Independent t-test was performed for normally-distributed variables, Mann-Whitney U test for non-parametric variables, and chi-squared test for categorical variables. p<0.05 is considered statistically significant.

**Abbreviations:** CNFD, corneal nerve fibre density; CNBD, corneal nerve branch density; CNFL, corneal nerve fibre length.
