# Supplementary material for: Male sexual dysfunction in obesity: The role of sex hormones and small fibre neuropathy
Source: PLoS One. 2019 Sep 11;14(9):e0221992. doi: 10.1371/journal.pone.0221992 (PMC6738611; doi:10.1371/journal.pone.0221992)
Supplement: S3 Table — Notes: Data are presented as mean and standard deviation for normally-distributed variables and median and interquartile range for non-parametric variables. Independent t-test was performed for normally-distributed variables, Mann-Whitney U test for non-parametric variables, and chi-squared test for categorical variables. p<0.05 is considered statistically significant. Abbreviations: CNFD, corneal nerve fibre density; CNBD, corneal nerve branch density; CNFL, corneal nerve fibre length. (DOCX) [file pone.0221992.s003.docx]

**S3 Table**. Comparison of sex hormone levels between symptomatic and asymptomatic patients divided based on **frequency of morning erections**.

|  | Asymptomatic based on frequency of morning erections  (n=6) | Symptomatic based on frequency of morning erections  (n=23) | *P*-value |
| --- | --- | --- | --- |
| Clinical characteristics |  |  |  |
| Age, years | 52.0±9.2 | 47.8±10.8 | 0.388 |
| Body mass index, kg/mm^2^ | 47.6±6.3 | 51.2±11.3 | 0.460 |
| Type 2 diabetes, n (%) | 5 (83%) | 14 (61%) | 0.303 |
| Duration of diabetes, years | 5±4 | 6±4 | 0.663 |
| Hypertension, n (%) | 4 (67%) | 11 (48%) | 0.411 |
| Biochemistry |  |  |  |
| HbA1c, mmol/mol | 67±15 | 49±11 | **0.033** |
| Total cholesterol, mmol/l | 3.7±0.4 | 4.0±1.1 | 0.662 |
| Triglyceride, mmol/l | 1.5±0.6 | 1.2±0.5 | 0.357 |
| HDL-C, mmol/l | 0.98±0.41 | 0.97±0.23 | 0.918 |
| LDL-C, mmol/l | 2.2±0.2 | 2.5±0.9 | 0.598 |
| Sex hormones |  |  |  |
| Low testosterone, n (%) | 3 (50%) | 14 (61%) | 0.630 |
| Total testosterone, nmol/L | 8.8 (6.9–13.6) | 9.0 (6.2–10.8) | 0.655 |
| Free testosterone, pmol/L | 193 (166–305) | 170 (116–235) | 0.278 |
| Sex hormone-binding globulin, nmol/L | 24.7 (15.6–39.6) | 31.8 (22.0–37.6) | 0.302 |
| Luteinising hormone, mIU/mL | 2.8±2.1 | 3.1±1.7 | 0.747 |
| Follicle-stimulating hormone, mIU/L | 3.2±2.1 | 3.7±1.4 | 0.501 |
| Dihydrotestosterone, nmol/L | 0.61 (0.39–1.03) | 0.60 (0.50–0.85) | 0.813 |
| Dehydroepiandrosterone sulphate, nmol/L | 2.2 (1.1–3.4) | 1.2 (0.8–3.9) | 0.059 |
| Androstenedione, nmol/L | 2.4 (1.6–3.1) | 1.6 (1.3–2.2) | 0.854 |
| CNFL, mm/mm^2^ | 19.27±3.58 | 17.76±4.54 | 0.465 |
| CNFD, no./mm^2^ | 28.97±3.51 | 26.46±6.49 | 0.376 |
| CNBD, no./mm^2^ | 47.95±23.51 | 52.55±30.71 | 0.739 |

**Notes:** Data are presented as mean and standard deviation for normally-distributed variables and median and interquartile range for non-parametric variables. Independent t-test was performed for normally-distributed variables, Mann-Whitney U test for non-parametric variables, and chi-squared test for categorical variables. p<0.05 is considered statistically significant.

**Abbreviations:** CNFD, corneal nerve fibre density; CNBD, corneal nerve branch density; CNFL, corneal nerve fibre length.
