# Supplementary material for: Male sexual dysfunction in obesity: The role of sex hormones and small fibre neuropathy
Source: PLoS One. 2019 Sep 11;14(9):e0221992. doi: 10.1371/journal.pone.0221992 (PMC6738611; doi:10.1371/journal.pone.0221992)
Supplement: S4 Table — Data are presented as median and interquartile range for non-parametric variables. Mann-Whitney U test was performed for non-parametric variables. Questionnaire response categories: erectile function: 1: always able to keep erection good enough for sexual intercourse, 2: usually able, 3: sometimes able, 4: never able; frequency of sexual thoughts and morning erection frequency: 1: none or once in the past month, 2: 2–3 times/month and 1 time/week, 3: 2–6 times/week, 4: ≥1/day; overall satisfaction: 0: very dissatisfied, 1: moderately dissatisfied, 2: equally satisfied and dissatisfied, 3: moderately satisfied, 4: very satisfied. Overall sexual function score ranges from 0 to 33 with higher scores corresponding with higher level of sexual functioning. Sexual functioning-related distress ranges from 0 to 20, with higher scores corresponding with higher level of distress. p<0.05 is considered statistically significant. (DOCX) [file pone.0221992.s004.docx]

**S4 Table**. Comparison of sexual symptoms between groups with low testosterone and normal testosterone.

|  | Normal testosterone  (n=12) | Low testosterone  (n=17) | *P*-value |
| --- | --- | --- | --- |
| Erectile function | 3 (1–4) | 3 (2–4) | 0.711 |
| Frequency of sexual thoughts | 3 (1–5) | 3 (0–5) | 0.811 |
| Frequency of morning erections | 0 (0–2) | 0 (0–1) | 0.845 |
| Overall sexual function | 10 (4–15) | 10 (0–16) | 0.777 |
| Sexual functioning-related distress | 3 (2–11) | 6 (2–10) | 0.499 |
| Overall satisfaction | 1 (0–2) | 0 (0–2) | 0.556 |

Data are presented as median and interquartile range for non-parametric variables. Mann-Whitney U test was performed for non-parametric variables.

Questionnaire response categories: erectile function: 1: always able to keep erection good enough for sexual intercourse, 2: usually able, 3: sometimes able, 4: never able; frequency of sexual thoughts and morning erection frequency: 1: none or once in the past month, 2: 2–3 times/month and 1 time/week, 3: 2–6 times/week, 4: ≥1/day; overall satisfaction: 0: very dissatisfied, 1: moderately dissatisfied, 2: equally satisfied and dissatisfied, 3: moderately satisfied, 4: very satisfied.

Overall sexual function score ranges from 0 to 33 with higher scores corresponding with higher level of sexual functioning.

Sexual functioning-related distress ranges from 0 to 20, with higher scores corresponding with higher level of distress.

p<0.05 is considered statistically significant.
